# ZIP4H (TEX11) Deficiency in the Mouse Impairs Meiotic Double Strand Break Repair and the Regulation of Crossing Over

**DOI:** 10.1371/journal.pgen.1000042

**Published:** 2008-03-28

**Authors:** Carrie A. Adelman, John H. J. Petrini

**Affiliations:** 1 Molecular Biology and Genetics Program, Sloan-Kettering Institute, New York, New York, United States of America; 2 Weill-Cornell Graduate School of Medical Science, New York, New York United States of America; Stowers Institute for Medical Research, United States of America

## Abstract

We have recently shown that hypomorphic Mre11 complex mouse mutants exhibit defects in the repair of meiotic double strand breaks (DSBs). This is associated with perturbation of synaptonemal complex morphogenesis, repair and regulation of crossover formation. To further assess the Mre11 complex's role in meiotic progression, we identified testis-specific NBS1-interacting proteins via two-hybrid screening in yeast. In this screen, *Zip4h* (*Tex11*), a male germ cell specific X-linked gene was isolated. Based on sequence and predicted structural similarity to the *S. cerevisiae* and *A. thaliana* Zip4 orthologs, ZIP4H appears to be the mammalian ortholog. In *S. cerevisiae* and *A. thaliana*, Zip4 is a meiosis-specific protein that regulates the level of meiotic crossovers, thus influencing homologous chromosome segregation in these organisms. As is true for hypomorphic *Nbs1* (*Nbs1^ΔB/ΔB^*) mice, *Zip4h^−/Y^* mutant mice were fertile. Analysis of spermatocytes revealed a delay in meiotic double strand break repair and decreased crossover formation as inferred from DMC1 and MLH1 staining patterns, respectively. Achiasmate chromosomes at the first meiotic division were also observed in *Zip4h^−/Y^* mutants, consistent with the observed reduction in MLH1 focus formation. These results indicate that meiotic functions of Zip4 family members are conserved and support the view that the Mre11 complex and ZIP4H interact functionally during the execution of the meiotic program in mammals.

## Author Summary

The process of meiosis is initiated by the formation of programmed DNA double strand breaks. The subsequent repair of these breaks is critical to the formation of egg and sperm in mammals. Numerous proteins that function in repair in somatic cells also have essential activities during meiotic repair. Examples of such proteins include the Mre11 complex, which is a detector of double strand breaks and plays a role in promoting their repair, as well as the kinase ATM, which governs cell cycle checkpoint responses in somatic cells. In this study, we report the isolation of a human, testis-specific Mre11 complex interacting protein, ZIP4H, and the establishment of a ZIP4H-deficient mouse. Sequence similarity, and phenotypic characterization of mice harboring a mutation in this gene indicate it is the mammalian ortholog of the meiosis-specific Zip4 proteins of *S. cerevisiae* and *A. thaliana*. Like its budding yeast and plant counterparts, mouse ZIP4H is required for efficient crossover formation between homologs. In the future, understanding the relationship between the Mre11 complex and ZIP4H will provide new insight to how Mre11 complex activities are adapted to the specialized framework of meiotic double strand break repair.

## Introduction

Meiosis is a specialized cell division program by which diploid organisms produce haploid gametes. This process of reductional division requires establishment of reciprocal exchanges, or crossovers, between homologous chromosomes. Crossovers arise from homologous recombination-mediated repair of programmed DNA double strand breaks. Crossover formation produces chiasmata, the physical connection between homologs which, in conjunction with sister chromatid cohesion, allows for biorientation of homologs at the first meiotic division [for review see 1],[2],[3]. Defects in this process can lead to the production of aneuploid gametes and the attrition of precursors during gametogenesis, both underlying causes of infertility [4]–[7],[and reviewed in 8],[9].

The Mre11 complex, consisting of MRE11, RAD50 and NBS1, is intimately involved in several aspects of the DSB response [for review see 10],[11], including break sensing [12]–[14], checkpoint activation [15]–[17], repair [18],[19], and the promotion of apoptosis [14],[20]. These activities also extend to meiotic DSB repair [reviewed in 1],[21], where it is variously employed in eukaryotes for initiation of meiotic DSBs [22],[23], processing of SPO11 from break ends [24],[25] (the enzyme that catalyzes meiotic breaks but remains covalently attached to DNA), checkpoint activation [17] and repair [26]–[28]. Because it is required for efficient DSB formation and for subsequent removal of Spo11 from break ends (for example in *S. cerevisiae* [22],[23] and *C. elegans* [29]), the functions of the Mre11 complex downstream of these events are less clearly understood.

We recently characterized the meiotic phenotypes of mice carrying hypomorphic mutations in *Mre11* and *Nbs1* (*Mre11^ATLD1/ATLD1^* [12] and *Nbs1^ΔB/ΔB^* [30] mice). These studies indicated that the complex is required for timely meiotic repair, synaptonemal complex morphogenesis and integrity, and accordingly, for regulating the formation of crossovers [31]. Given the global involvement of the Mre11 complex in chromosome break metabolism, we reasoned that meiosis-specific functional interactions would be required to situate the complex within the meiotic program. We carried out a yeast two-hybrid screen of a human testis cDNA library. The C-terminus of a spermatogonial-expressed X-linked gene, TEX11 [32], was among the proteins isolated using NBS1 as bait. As *Tex11* expression was reported to be spermatogonia-specific [32], we undertook phenotypic analysis of TEX11-deficient mice.

Mammalian TEX11 proteins exhibit similarity to *A. thaliana* and *S. cerevisiae* Zip4. Phenotypic characterization of Zip4 deficiency in these organisms indicates the respective gene products have meiotic functions. The Zip4 orthologs are required for formation of the bulk of interfering (or class I) crossovers during *S. cerevisiae* [33] and *A. thaliana* meiosis [34]. In *S. cerevisiae*, but not *A. thaliana*, the protein is also required for morphogenesis of the synaptonemal complex (SC), a proteinacious structure that joins homologous chromosomes in close proximity during meiotic repair [reviewed in 35]. Impairment of these functions results in meiosis I (MI) segregation defects, leading to aneuploidy and spore inviability in *S. cerevisiae*, and sterility in *A. thaliana*.

The phenotypes of TEX11-deficient mice are consistent with the interpretation that TEX11 is the mammalian Zip4 ortholog, and thus will heretofore be referred to as ZIP4 homolog, or ZIP4H. ZIP4H-deficient spermatocytes exhibit delayed resolution of DSB repair intermediates and impaired regulation of crossing over, resulting in production of achiasmate chromosomes at the MI division. Although crossover perturbations were not as severe as those seen in *S. cerevisiae* and *A. thaliana zip4* mutants, these results indicate the meiotic functions of Zip4 orthologs have been conserved. Furthermore, the phenotypic similarity of ZIP4H and Mre11 complex mutant mice with respect to delayed resolution of repair intermediates [31], and the physical interaction between NBS1 and ZIP4H suggest a collaborative function for these proteins during the meiotic DSB response.

## Results

Components of the general DNA damage response influence the execution of the meiotic program in concert with meiosis specific factors. Having previously assessed the meiotic phenotypes of hympomorphic Mre11 complex mutants in the mouse [31], we carried out a yeast two-hybrid screen of a human testis cDNA library to identify meiosis-specific factors that interact with the Mre11 complex using NBS1 as the bait. The C-terminus of ZIP4H was among the proteins isolated in this screen.

**Figure 1:**
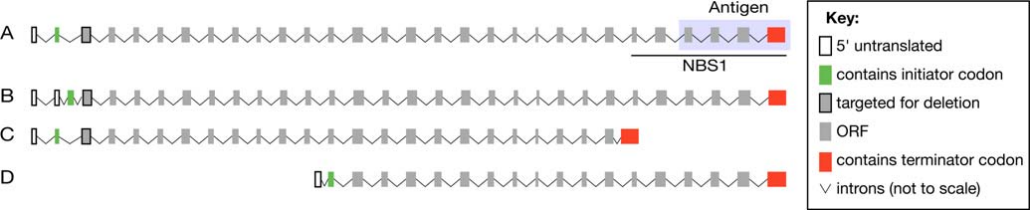
Mammalian *Zip4h* transcripts. Alignment of *Zip4h* isoforms retrieved from public sequence databases indicate similar full-length isoforms are present from human and mouse testis extracts (A); accession #'s NM_031276 and NM_031384, respectively. A second human isoform isolated from testis contains a novel exon inserted between exons 2 and 3 of the full-length isoform (B); accession # NM_001003811. A C-terminally truncated transcript with an alternate terminal exon spliced to exon 24 was found in mouse ES cells (C); accession # AK082794. An N-terminally truncated isoform containing a novel 5′ untranslated exon spliced to exon 13 was isolated from human testis (D); accession # AK057391. Exons corresponding to ZIP4H fragment isolated in the yeast two hybrid screen (NBS1) and the antigen used for antibody generation (Antigen) are illustrated in A.

Alignment of Zip4 orthologs revealed relatively modest homology at the primary sequence level [see Chelysheva et al. 34]; however sequence queries against protein domain databases indicated the presence of multiple predicted TPR motifs (protein interaction domains, reviewed in [36]) in all species, suggesting significant structural homology between ZIP4H and previously identified Zip4 orthologs (data not shown, and see Perry et al. [37]). The presence of at least 4 mouse and human isoforms was noted (Figure 1A–D) which include isoforms with species-specific exons (Figure 1B and 1D) and C- and N-terminally truncated isoforms of murine and human *Zip4h*, respectively (Figure 1C and 1D). With the exception of the C-terminally truncated form expressed in embryonic stem cells (Figure 1C), all isoforms were reportedly isolated from testis. We verified the presence of the full length and C-terminally truncated forms (Figure 1A and 1D) in cDNA generated from mouse testis and embryonic stem cells (not shown).

Northern blot analysis of human tissues confirmed that *Zip4h* mRNA was restricted to testis (Figure S1A). Consistent with previously published results [32], *Zip4h* was not expressed in adult ovaries. To address whether *Zip4h* was expressed in oogonia or oocytes we isolated ovaries from embryonic day 15.5 mice, the stage when most oocytes are in the leptotene and zygotene stages of meiosis [38, see Text S1 for definition of meiotic stages]. RT-PCR analysis indicated the presence of *Zip4h* transcripts in these preparations (data not shown).

**Figure 2:**
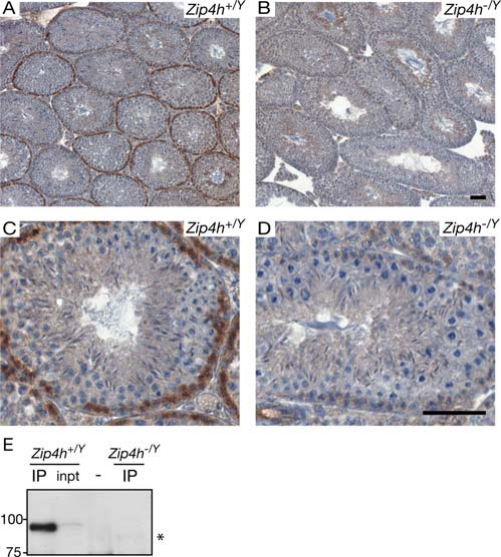
Zip4h expression in testis sections. Testis sections from adult *Zip4h^+/Y^* (A, C) and *Zip4h^−/Y^* (B, D) mice were immunohistochemically labeled for ZIP4H (in brown), and counterstained with hematoxylin (in blue). Staining patterns under low magnification (A, B). Higher magnification images showing positively stained zygotene cells of control tubules (C) and absence of staining in mutant tubules at the same stage (D). Black bars represent 50 µm. ZIP4H IP-Western blot from *Zip4h^+/Y^* and Z*ip4h^−/Y^* 12 d.p.p. testis extracts (E), asterisk denotes lower molecular weight species visible only in *Zip4h^−/Y^* samples.

To determine the localization of ZIP4H protein, antiserum was raised against mouse ZIP4H in goat and rat using an antigen spanning the region of the protein encoded by the last 4 exons (see Figure 1A for illustration). Immunohistochemical staining of testis sections from wildtype mice indicated ZIP4H-positive cells were localized to the periphery of a subset of tubules (Figure 2A and 2C). Staging of ZIP4H positive tubules and cells [39] indicated that the protein appears in late stage spermatogonia and pre-meiotic cells, is present through the early meiotic stages of leptotene and zygotene, and diminishes to background levels during pachytene (Figure S2, Table S1). Immunofluorescence analysis of spermatocyte spreads using four different ZIP4H antisera did not reveal areas of concentrated ZIP4H localization (data not shown) indicating that either ZIP4H does not localize to discrete sites along SCs or chromatin domains such as the sex body, or enrichment at these sites is insufficient for detection. In this regard, it is noteworthy that with the exception of the sex body, the Mre11 complex exhibits a similar lack of focal presentation on meiotic chromatin [40]. Nevertheless, as also shown for the Mre11 complex, immunohistochemical analyses indicated that ZIP4H is present during meiotic stages when key events take place including DSB formation, repair, synaptonemal complex assembly, and early phases of crossover site establishment.

### *Zip4h* Targeting and Deletion in the Mouse

To determine the function of ZIP4H in mammals, we used gene targeting to derive ZIP4H-deficient mice (Figure S1B–D). Exon 3, the first exon common to three of the isoforms, was selected for deletion. Proper configuration of the targeted locus was confirmed via Southern blot (Figure S1E and S1F). ES cells carrying the floxed allele were transfected with a *Cre* expression construct to determine the effect of exon 3 deletion. RT-PCR and sequencing of the PCR product indicated that the message produced in *Zip4h^−/Y^* ES cells contained an exon 2 to 4 splice product (Figure S1G–J), with the resulting frameshift creating a premature termination codon 6 nucleotides down stream. The *Zip4h^ind/Y^* ES cells were used to generate chimeras, and mice harboring the conditional allele (*Zip4h^ind^*) were obtained and mated with *Cre* transgenic mice [41] to produce the deleted allele (*Zip4h^−^*, see Figure S1K for genotyping results). *Zip4h^−^* mice were fertile, and produced normal-sized litters with normal Mendelian ratios of mutant males and females.

Immunohistochemical analysis of testis sections indicated that in comparison to littermate controls, ZIP4H protein appeared absent in the *Zip4h^−/Y^* mutants (Figure 2B and 2D). Analysis of testis lysates was also carried out. As ZIP4H is present at very low levels in testis extract, immunoprecipitation is required for detection. ZIP4H was immunoprecipitated from testis lysates prepared from 12-day post partum (d.p.p.) males. At this age, germ cells are undergoing the semi-synchronous first wave of spermatogenesis and are predominantly found in the pre-leptotene, leptotene and zygotene stages. In wildtype mice, a band migrating slightly faster than 100 kD was observed, similar to the predicted molecular weight of 109 kD (Figure 2E). This species was absent from extracts of *Zip4h^−/Y^* mice; however a faint band of lower molecular weight was visible (see Figure 2E asterisk), raising the possibility that the allele created may produce a variant protein product, albeit at a markedly reduced level.

### DSB Repair and Crossing Over is Perturbed in *Zip4h^−/Y^* Mice

**Figure 3:**
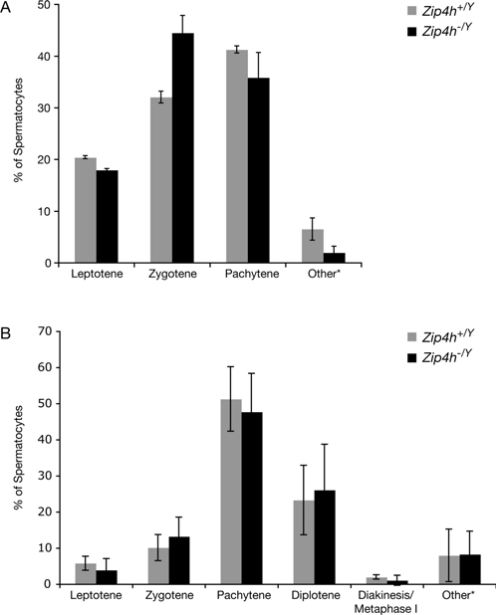
Meiotic stage distribution in mutant and control spermatocytes. Spermatocyte spreads from 12 d.p.p. (A) and adult mice (B) were stained with SCP3 and SCP1 to analyze SC morphogenesis, and categorized according to meiotic stage. *Other denotes extensively fragmented or otherwise unidentifiable cells.

Cytological preparations of *Zip4h^−/Y^* spermatocytes were examined for meiotic defects. In contrast to *S. cerevisiae zip4* mutants [33], spermatocytes from *Zip4h^−/Y^* mice did not exhibit significant perturbations in synaptonemal complex morphogenesis or integrity (Figure 3, Figure S3, and Table S2). There was a subtle increase in percentage of zygotene cells during the semi-synchronous first wave of meiosis in juvenile males (Figure 3A, χ^2^ = 19.3, P = 0.00024). This same trend was suggested in the asynchronous adult population of spermatocytes, but the difference was not significant (Figure 3B, χ^2^ = 5.38, P = 0.37). Since pachytene cells forming crossovers always appeared completely synapsed, it appears that although SC morphogenesis is subtly perturbed at early stages of meiosis, this does not prevent cells from ultimately completing synapsis. These results are similar to *A. thaliana zip4* mutants in which synapsis initiation appears to be decreased, but the completion of synapsis is not abolished [34].

**Figure 4:**
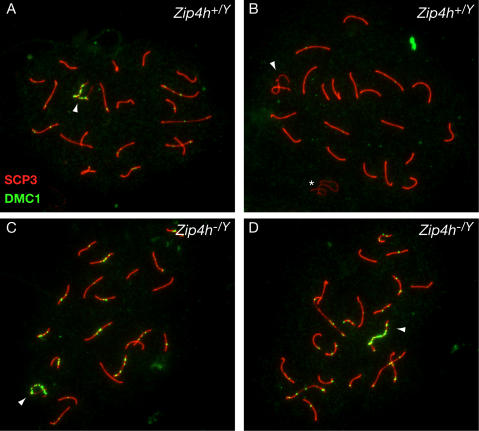
Persistent DSB repair intermediates in pachytene cells of *Zip4h^−/Y^* mice. Spermatocyte spreads prepared from adult *Zip4h^+/Y^* and *Zip4h^−/Y^* mice were stained for DMC1 (green) and SCP3 (red). Representative images of pachytene stage spermatocytes from wildtype (A, B) and mutant (C, D) mice are shown. XY bivalents are indicated by arrowheads; asterisk in B indicates XY bivalent from adjacent spread.

The RecA paralogs RAD51 and DMC1 catalyze strand exchange during meiotic DSB repair [42]–[44]. Using antisera that recognize RAD51 and DMC1, we previously established evidence that the repair of meiotic DSBs is delayed in *Nbs1^ΔB/ΔB^* and *Mre11^ATLD1/ATLD1^* mice [31]. To determine whether a similar defect was present in *Zip4h^−/Y^* mice, DSB repair intermediates were immunofluorescently labeled with DMC1 antibody and the SC marker, SCP3. We observed an increase in DMC1 foci in pachytene cells of adult *Zip4h^−/Y^* mice. These cells contained 51.4 DMC1 foci on average (42.1 autosomal and 9.2 XY foci), whereas controls contained 20.0 foci on average (14.6 autosomal and 5.6 XY foci; Table 1 and Figure 4; Wilcoxan rank sum, W = 7499.5, P(two-sided) = 8.43e-10). This difference did not appear to reflect differences in DSB formation or early events in DSB processing since average DMC1 foci numbers in leptotene and early zygotene spermatocytes from 12 d.p.p. juvenile males were comparable to controls (Table 1; W = 1507, P(two-sided) = 0.90 and W = 1375.5, P(two-sided) = 0.63, respectively). Pachytene spermatocytes from juvenile mice mirrored the adult situation; mutants exhibited significantly higher mean DMC1 foci numbers (Table 1; W = 1125, P(two-sided) =  1.90e-6). Together, these results suggest that meiotic DSBs are formed and processed normally in the mutants, but repair intermediates are not resolved in a timely fashion.

**Table 1:** Average DMC1 Foci

Genotype	Juvenile^1^			Adult^2^
	Leptotene	Early Zygotene^3^	Pachytene	Pachytene
	Mean (SD)	Mean (SD)	Mean (SD)	Mean (SD)
*Zip4h^+/Y^*	202 (46)	212 (39)	46.6 (20.3)	20.0 (23.4)
*Zip4h^−/Y^*	210 (69)	206 (39)	67.8 (19.6)	51.4 (35.9)

1 More than 35 cells examined per stage, 2 mice per genotype;

2 more than 94 cells examined, 3 mice per genotype;

3 Zygotene cells with less than 3 clear bivalents formed.

**Figure 5:**
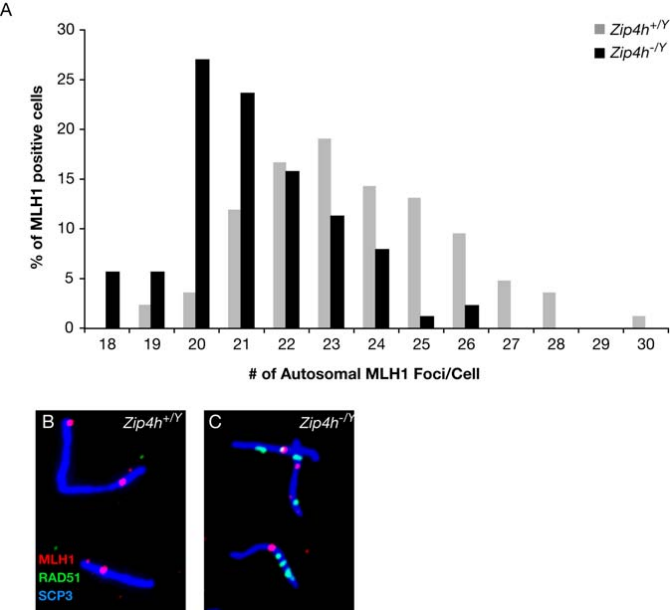
*Zip4h^−/Y^* mice exhibit perturbations with respect to crossover formation. Numbers of MLH1 foci per cell were tabulated in MLH1 positive spermatocyte spreads from *Zip4h^+/Y^* and *Zip4^−/Y^* mice and percent spreads containing the indicated foci were graphed (A). To determine whether DSB repair foci persisted to the stage when crossovers form, cells were co-stained with antibodies against MLH1 (red), RAD51 (green) and SCP3 (blue). Representative bivalents from *Zip4h^+/Y^* (B), and *Zip4h^−/Y^* (C) spreads are shown.

To determine if ZIP4H deficiency influenced crossover formation, spermatocytes were labeled with MLH1 antibody. MLH1 is a MutL family protein required for formation of the bulk of crossovers in mice, and MLH1 foci numbers and distribution closely correlate with those of crossovers [45]–[48]. Although the small subset of crossovers formed via MLH1-independent mechanisms [4],[45],[49] are not addressed in these analyses, the number and disposition of the majority of crossovers can be assessed by MLH1 staining. A significant decrease in mean autosomal crossovers was observed, from 23.5 in controls to 21.2 in the mutants (Wilcoxan rank sum, W = 9505.5, P(two-sided) = 1.60e-11; Figure 5A). It is noteworthy that because the minimum threshold was set at 18 foci, this value likely under represents the reduction in crossovers in the mutants. Perhaps due to the transient association of MLH1 at the XY crossover, measurement of XY bivalent exchange frequencies was highly variable in both mutants and controls, and therefore inconclusive (for example, percent wildtype bivalents with 0 crossovers: autosomes = 1.25%±0.5 SD; XY = 82%±14.5). Since percentage of MLH1 positive pachytene cells was similar (35% in controls and 33% in mutants), and MLH1 positive diplotene cells were rarely observed, these results imply that recombination is reduced in the absence of ZIP4H, rather than delayed.

Normally, the majority of DSB repair intermediates are resolved before crossover formation ensues [50]. To determine the relationship between DSB repair intermediates and crossovers, pachytene spermatocytes were co-stained with RAD51 and MLH1 (RAD51 results were similar to that of DMC1; not shown). 36% of autosomes (72/200) in *Zip4h^−/Y^* pachytene cells were decorated with both RAD51 and MLH1 foci, whereas only 12% of control autosomes (27/220) exhibited this pattern (Figure 5B and 5C). These foci also corresponded to sites of γ-H2AX labeling (Figure S4), confirming they marked unrepaired breaks. Therefore, instances where crossover formation is concurrent with ongoing repair are increased in the mutant, further supporting the proposal of delayed or defective repair in these mice.

**Figure 6:**
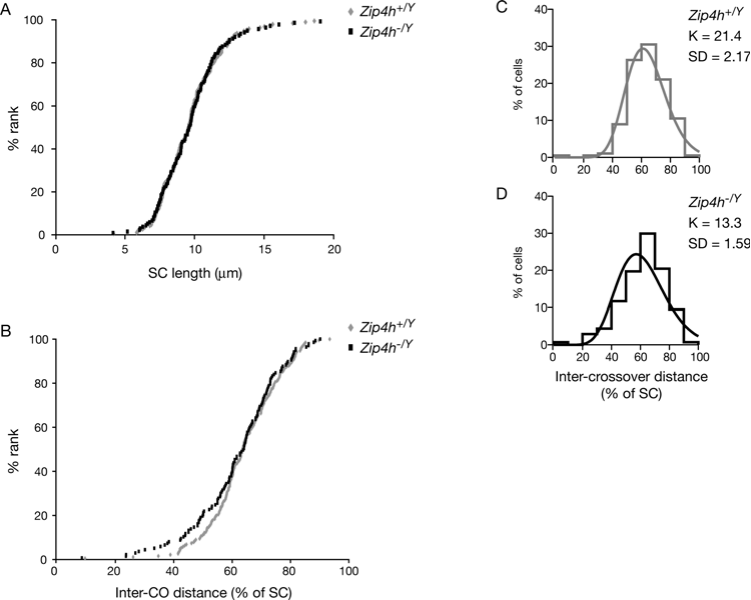
Crossover interference is reduced in *Zip4h* mutants. SC lengths (A) and inter-crossover distance expressed as % of SC (B) of double exchange bivalents in *Zip4h^+/Y^* and *Zip4h^−/Y^* spermatocytes are graphed according to increasing length. Maximum likelihood fitting of the data to the gamma-distribution allows estimation of the interference parameter, K, for control (C) and mutant (D) double exchange bivalents.

Interference describes the fact that crossovers form non-randomly such that formation of closely spaced crossovers is disfavored [51],[52]. The reduction in crossovers noted in *Zip4h^−/Y^* mutants, as well as apparent abolishment of interfering crossovers in *S. cerevisiae* and *A. thaliana zip4* mutants [33],[34] led us to examine crossover interference in *Zip4h^−/Y^* mice. Consistent with the observation of reduced crossovers, there were approximately half as many double exchange bivalents in *Zip4h^−/Y^* spreads as in *Zip4h^+/Y^* (25%±3.3 versus 13%±1.5, respectively). Measurement of SC length and inter-MLH1 distance was carried out on the residual double crossover bivalents (minimum of 137 bivalents per genotype). Since interference can vary between different chromosomes [53], we compared the SC lengths of double exchange bivalents in wildtype and mutants. This comparison demonstrated that SC lengths did not vary as a function of genotype (Figure 6A). The implication of this finding is that the occurrence of double exchange bivalents in *Zip4h^−/Y^* spermatocyte spreads is not restricted to a particular SC length class; therefore data describing inter-crossover distances in wild type and *Zip4h^−/Y^* are comparable.

Using maximum likelihood fitting of inter-crossover distances to the gamma-distribution, the estimated shape value reflects the strength of interference [54]–[56]. Using this method, samples exhibiting no interference (and thus high variance in inter-focus distance) fit best to curves having a shape value, or interference parameter, of 1. As samples exhibiting increasing interference are analyzed, the best-fit curve narrows as a result of decreasing variance in inter-focus distance, and the interference parameter in turn increases. The interference parameter estimate for wildtype samples is 21.4 (SD = 2.17, rate = 0.33), whereas for mutants it is 13.3 (SD = 1.58, rate = 0.22, [57], see Figure 6C and 6D).

To compare wildtype and mutant interference patterns the measurements were graphed in cumulative distribution curves (Figure 6B). This indicated the mutant distribution was skewed toward lower inter-crossover distances. In order to test whether these data differ significantly from the wildtype we utilized the following strategy: a table was constructed in which the proportion of SCs with inter-crossover distances spanning less than 10%, 20%, 30%, and so on, of the total SC length was calculated for each group. Since crossovers tend to be widely spaced, perturbations in interference should become especially evident with respect to the proportion of SCs containing lower inter-crossover distance to SC length ratios. From this table it was apparent the proportion of SCs falling in the categories between 10 and 50% was consistently higher in the mutants than in controls, and this difference was statistically significant for both the inter-crossover distances falling below 40% and 50% of total SC length (e.g.: 11% of control inter-crossover distance measurements span less than 50% of the SC versus 20% in mutants; χ^2^ = 4.762, P = 0.029). These data indicate that crossovers in ZIP4H-deficient mice still exhibit strong interference but the mutants have a higher proportion of double-exchange SCs with short inter-crossover distances, indicating that interference is weaker than in controls.

### Achiasmate Chromosomes in Metaphase I Cells from *Zip4h^−/Y^* Mice

**Figure 7:**
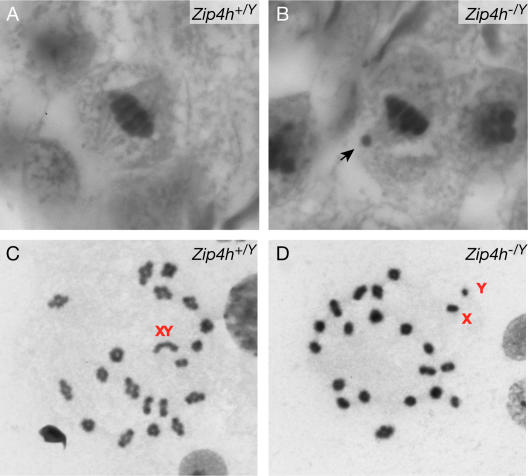
Increased achiasmate chromosomes in MI cells from *Zip4h^−/Y^* mice. Metaphase I spindles from stage XII tubules of *Zip4h^+/Y^* (A) and *Zip4h^−/Y^* (B) sections. Arrow indicates laggard. Representative diakinesis spreads from a wildtype mouse with no achiasmate chromosomes (C), and a mutant mouse exhibiting achiasmate XY chromosomes (D).

The decreased crossover frequencies indicated by MLH1 analyses raised the possibility that cells progress to metaphase with non-crossover products. This would in turn lead to univalent chromosomes as a sequela of crossover defects. To test this prediction, MI spindles were examined in histologically stained testis sections. A significant increase in the proportion of *Zip4h^−/Y^* spindles exhibiting laggard chromosomes was observed: from 5.6% (11/196) in wildtype, to 12.9% (19/147) in mutants (Figure 7A and 7B; Mantel-Haenszel, M = −2.306, P(one-sided) = 0.010). To determine whether laggards were in fact achiasmate chromosomes, diakinesis spreads were prepared. *Zip4h^−/Y^* samples exhibited an increase in both achiasmate XY chromosomes and autosomes (11% and 14%, respectively) compared with controls (1.2% and 9.9%, respectively; Figure 7C and 7D). In total, 25% (18/71) of mutant diakinesis spreads contained achiasmate chromosomes, versus 11% (9/81) of controls (Mantel-Haenszel, M = 2.35, P(one-sided) = 0.0095). These results indicate that reduced crossover formation in *Zip4h^−/Y^* spermatocytes results in progression to the MI division in the presence of achiasmate chromosomes.

To assess the fate of MI cells with achiasmate chromosomes, the frequency of aneuploidy was assessed in metaphase II (MII) chromosome spreads. Both genotypes had the same frequency of aneuploid cells containing less than 20 chromosomes (30% in wildtype and 25% in mutants). In contrast, no metaphase spreads contained more than 20 chromosomes (60 MII cells analyzed from mutants, and 46 from controls); therefore it is likely that cells with less than 20 chromosomes largely represent cells that have artifactually lost chromosomes during preparations of the spreads.

**Figure 8:**
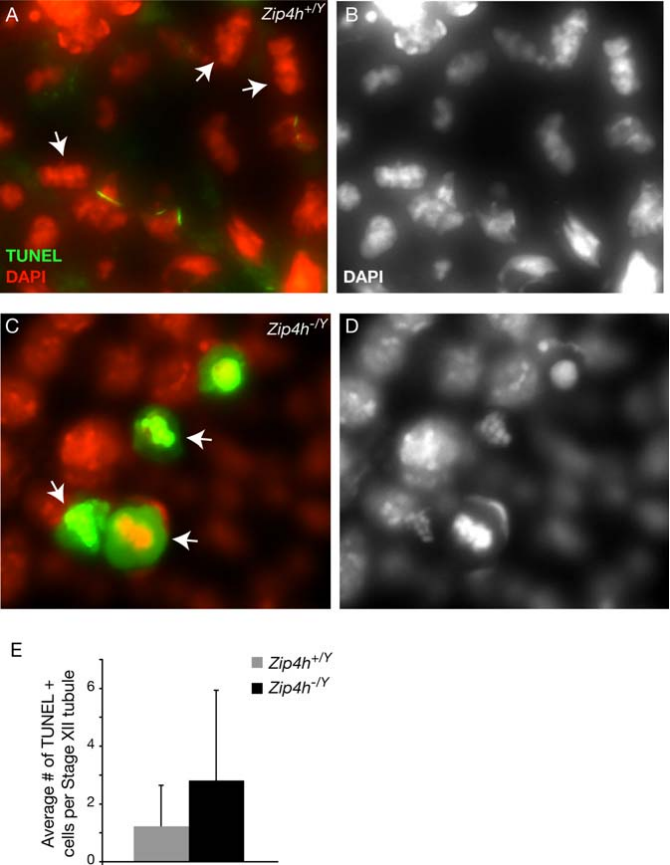
TUNEL positive metaphase cells in testis of *Zip4h^−/Y^* mice. Testis sections from mutant and control mice were TUNEL labeled (green) and counterstained with DAPI (pseudocolored red) to assess cell death. TUNEL negative stage XII section from a wildtype mouse (A, B) containing cells with metaphase configurations (A, arrows; same image in B showing only greyscale DAPI). TUNEL positive cells from a *Zip4h^−/Y^* mouse (C, D), some exhibiting metaphase configurations (arrows). Average numbers of TUNEL positive cells per stage XII tubule are graphed with standard deviations (E).

To determine if increased cell death was apparent in tubules in the meiotic division stage (stage XII) of the seminiferous cycle, testis sections were TUNEL stained and analyzed. The number of TUNEL positive cells in stage XII tubules increased more than two fold, from 1.2 in controls to 2.8 in *Zip4h* mutants (Figure 8; Wilcoxan, W = 681, P(one sided) = 0.03). These cells often had a hypercondensed metaphase-like configuration of chromatin (see Figure 8C and 8D). Although no significant difference was observed in the overall proportion of TUNEL positive cells when all tubules were analyzed, this may be explained via feedback and integration of death at this stage into the mechanisms that regulate sperm production and cell death throughout the spermatogenic cycle [58]–[60]. It is not clear whether TUNEL positive MI cells represent true apoptotic cells, however death of mitotically arrested cells has been reported previously [4],[61],[62]. This result correlates with the magnitude of the increase in diakinesis spreads containing achiasmate chromosomes, and is consistent with the lack of aneuploid MII metaphases. We propose that these observations are a result of achiasmate chromosomes triggering a spindle checkpoint [61] at MI leading to cell death.

## Discussion

In these studies we characterize the phenotypes arising from mutation of the spermatogonia-expressed gene, *Zip4h*. We show that ZIP4H-deficiency impairs timely repair of meiotic DSBs. Similar to Zip4 orthologs from other organisms, the data presented further demonstrate that crossover formation is impaired in ZIP4H-deficient mice resulting in achiasmate chromosomes at MI.

### Zip4 Proteins in Meiotic Synapsis, Repair and Crossover Formation

Studies of Zip4 deficiency in *S. cerevisiae* revealed that both synapsis and crossover formation were impaired, whereas no defect in noncrossover repair was observed [33]. In *A. thaliana zip4* mutants, no significant SC morphogenesis defects were observed. Conversely, crossover defects similar to budding yeast were reported [34]. In our studies, ZIP4H deficiency resulted in decreased crossovers without synapsis defects, suggesting that ZIP4H activities in mammals more closely resemble those of *A. thaliana* than *S. cerevisiae* Zip4 orthologs. Furthermore, increased DMC1 foci [34] and persistent DMC1 repair intermediates (this study) were observed in *Atzip4* and mouse *Zip4h* mutants, respectively, reflecting similar perturbations in repair.

In light of *S. cerevisiae* Zip4 functions and the apparent uncoupling of synapsis and repair in mouse *Zip4h* mutants, the possibility that the *Zip4h* allele established here is hypomorphic as opposed to null is raised. We observed a lower molecular weight species present at substantially reduced levels in comparison to wildtype ZIP4H in IP-Western blots from mutants (see asterisk in Figure 2E). In this scenario, ZIP4H's putative role in synapsis would be retained in the *Zip4h* allele reported here; thus undermining the view that mammalian and *S. cerevisiae* Zip4 are functionally divergent.

On the other hand, the similarity between mouse ZIP4H and *A. thaliana* Zip4 deficiency supports the view that crossover formation and synapsis may be uncoupled. In that case, Zip4's influence on crossovers would be directly related to crossover promotion rather than reflecting an indirect effect arising from SC assembly and DNA repair defects. This possibility is resonant with a number of observations: *Zip4* disruption in *A. thaliana* and *S. cerevisiae* specifically impairs formation of class I crossovers [33],[34], the primary crossover class in this and many other species [63]–[66]; *S. cerevisiae* Zip4 localization appears to coincide with crossover sites [33], [67]–[69]; and neither *Atzip4*, nor mouse *Zip4h* mutants show any indication that DSB induction or engagement of RAD51 and DMC1 is impaired in leptotene or zygotene (see Chelysheva et al. [34] and leptotene and zygotene DMC1 foci, Table 1), suggesting crossover perturbations do not stem from the impairment of early events such as DSB formation or processing. These observations point toward local Zip4 functions in promoting crossovers.

The presence of multiple TPR motifs in the Zip4 orthologs [37], which are likely to drive protein interactions [70], further supports the view that crossover promotion by Zip4 may be via recruitment of recombination factors. In this context, persistent DSB repair intermediates in *Zip4h* mutants may stem from deficient recruitment of either repair components, or factors required for RAD51 and DMC1 filament turnover. As chromosome fragments are not detected in division stage cells from *A. thaliana zip4* and the *Zip4h^−/Y^* mutant reported here, these intermediates are likely to be resolved via an alternate non-crossover mechanism or sister repair later in pachytene [71],[72].

The possibility that all class I crossovers are abolished in *S. cerevisiae* and *A. thaliana zip4* mutants [33],[34] suggests that a true assessment of Zip4's effects on class I crossover interference may be problematic in these organisms. This conclusion is based on the absence of interference between remaining crossovers in these mutants (characteristic of class II, MUS81/MMS4 dependent crossovers [63]), epistasis between *Atzip4* and *Atmsh4* mutants (the latter being solely involved in formation of class I, interfering crossovers [73],[74], and the observation that in *S. cerevisiae zip4-mms4* double mutants, all crossovers were abolished (indicating these genes act in separate crossover pathways). In contrast, mouse *Zip4h* mutants retain a large proportion of class I (i.e., MLH1-dependent) crossovers. Although these crossovers exhibit interference, their interference is reduced in comparison to controls. Since MLH1 is thought to mediate only class I, and not class II crossovers [75],[76], reduced interference in *Zip4h* mutant mice indicates a role for Zip4 orthologs in both class I crossover formation and interference.

### The Link Between Zip4 and the Mre11 Complex

The relationship between ZIP4H and the Mre11 complex has important implications with respect to the meiotic functions of these proteins and where they are situated in the meiotic program. The two-hybrid interaction between human ZIP4H and NBS1 points toward a collaborative relationship between these factors during meiosis. We used several approaches to further validate this interaction. Currently, we are unable to reliably coimmunoprecipitate ZIP4H and NBS1 from extracts of adult (asynchronous) or juvenile (leptotene/zygotene enriched) mouse testis. This is likely attributable to the relatively small pool of cells from whole testis, even in partially enriched fractions, in which *Zip4h* is expressed. That is, the bulk of ubiquitously expressed NBS1 is precipitated from cells not expressing ZIP4H. Moreover, the antibody used to precipitate ZIP4H was raised against an antigen falling in the NBS1 interacting region (see Figure 1A), perhaps explaining failure to precipitate NBS1 with ZIP4H. However, a small quantity of human NBS1 co-precipitated with transiently expressed human ZIP4H in 293T cells (see Figure S1L), and vice versa. If the Mre11 complex and ZIP4H are part of a larger meiotic damage response complex, the lack of additional meiotic factors in these mitotic cells may account for the apparent weakness of the interaction.

A number of observations in addition to the NBS1 ZIP4H interaction suggest the Mre11 complex and ZIP4H may collaborate during meiosis. The phenotypic outcomes of Mre11 complex hypomorphism and ZIP4H-deficiency are similar; for example, both exhibit persistence of DSB repair intermediates [31]. In *S. cerevisiae* the Mre11 complex accumulates at unprocessed meiotic breaks coinciding with Zip2 foci [77]–[79]. This, together with the observations that Zip2, Zip3, and Zip4 foci colocalize [33],[79], and Zip3 and Mre11 physically interact [79], suggest that the Mre11 complex and Zip4 are at least in spatial proximity during early stages of break processing or repair in budding yeast. Collectively, these results point toward an association between these proteins.

The failure of *Zip4h* mutants to phenocopy Mre11 complex hypomorphs in other respects is puzzling. Whereas both *Zip4h^−/Y^* and Mre11 complex hypomorphs exhibit DNA repair defects, SC morphogenesis and integrity is compromised in Mre11 complex hypomorphs [31], but not in *Zip4h* mutants. The lack of phenocopy between these mutants may indicate that synapsis defects and persistent DSB repair intermediates have distinct mechanistic bases in Mre11 complex hypomorphs, and that only the DNA repair facet of that phenotype is ZIP4H-dependent. In this regard, it is noteworthy that the persistence of DMC1 and RAD51 foci in *Nbs1^ΔB/ΔB^ Zip4h^−/Y^* double mutants is not additive with respect to the single mutants (data not shown).

As is true for Mre11 complex components, we do not see evidence of ZIP4H localization to discrete sites on meiotic chromatin, as would be expected if the protein associated with DSB sites. Indeed, the Mre11 complex appears to be ubiquitously associated with chromatin, and enriched in the sex body [40],[80]. ZIP4H localization in spermatocyte spreads using our antisera has been similarly uninformative, indicating the antibodies either do not recognize ZIP4H under these conditions, or the protein exhibits no apparent localized enrichments on meiotic chromatin. In *S. cerevisiae,* Zip4 localizes to discrete foci [33], which correlate with the number and distribution of crossover sites [67]–[69] suggesting that this Zip4 ortholog localizes to a specific subset of breaks. In mice, immunohistochemical staining reveals ZIP4H's presence during break initiation and metabolism stages (see Figure S2), similar to the stages when Mre11 complex components are upregulated [80 and our unpublished data]. In light of these observations, ZIP4H's interaction with NBS1, and the DSB associated activities of the Mre11 complex, localization of mammalian ZIP4H to at least a subset of meiotic breaks is plausible.

The role of the DNA damage response in effecting the meiotic program is clearly supported by the phenotypes of Mre11 complex mutants [27],[31],[81] as well as ATM [82]–[84], the transducing kinase it regulates. The connection reported here between the Mre11 complex and mammalian ZIP4H underscores a basic theme in meiotic DSB repair in which meiosis-specific proteins provide structural and interaction based frameworks in which non-meiosis-specific DSB response factors operate.

## Methods

### Yeast Two Hybrid Screening

Human NBS1 was expressed as a GAL4 DNA-binding domain fusion protein from pAS1 in the yeast strain, PJ69-4A [85]. A human testis cDNA library in pACTII (Clonetech) was screened for interactions with NBS1 by selecting for colony growth in the absence of adenine. Adenine prototrophic colonies were retested on plates lacking histidine or adenine, and pACTII cDNA clones were isolated from yeast exhibiting adenine and histidine prototrophy and analyzed by DNA sequencing.

### Mice

Mice were housed in ventilated rack caging in a pathogen-free facility. Animal use protocols were approved by the Institutional Animal Care and Use Committee of Memorial Sloan-Kettering Cancer Center. Mice were maintained on a mixed C57BL/6, CD-1, 129Sv background. For all experiments described herein, littermate mutant and control males were utilized.

### Generation of ZIP4H Antibodies and ZIP4H IP-Western Blotting

Goat-anti-mouse-ZIP4H and rat-anti-mouse-ZIP4H antibodies were generated at & D Systems using an antigen from the C-terminus of the protein encoded by the 4 terminal exons. For preparation of testis lysates for IP-Western blotting, testes from juvenile males between the ages of 12–14 d.p.p. were isolated and tubules disaggregated in D-MEM. Pelleted cells were extracted with lysis buffer (10 mM Tris, pH7.5, 5 mM EDTA, 0.5% NP-40, 250 mM NaCl) at 4° and cleared by centrifugation. Protein levels were quantified using the DC protein assay (BioRad), and immune complexes precipitated with protein G+A beads (Calbiochem) from 0.4 mg of lysate using goat-anti-mouse-ZIP4H (R & D Systems). Samples were run on 3–8% tris-acetate gels (Invitrogen) and transferred to PVDF (Millipore). Blots were incubated overnight with rat-anti-mouse-ZIP4H antibody (R & D Systems), probed with anti rat-HRP (Pierce), incubated with enhanced chemiluminescent substrate (Amersham), and exposed to film.

### Spermatocyte Preparation and Immunostaining

For meiotic studies, adult males between the ages of 8 and 16 weeks were utilized. For DMC1 analyses in juvenile males, pups were sacrificed 12 d.p.p. Testes were isolated and processed using a modified version of the surface-spread meiocyte preparations method described by Peters et al. [86]. Briefly, the tunica was removed from the testis and tubules disaggregated and chopped with razorblade in D-MEM. Aliquots of the cell solution were transferred to multi-well slides containing hypotonic buffer (0.5% NaCl, pH 8), and incubated 15 minutes to allow attachment of cells to the slide surface. Cells were fixed in 2% paraformaldehyde (PFA) with 0.03% SDS for 3 minutes, 2% PFA for an additional 3 minutes, and then rinsed and allowed to air dry for 10 minutes. Slides were incubated in 1x block (10x stock: 10% goat serum (Sigma), 3% BSA, 0.05% TX-100, in PBS), then stained overnight at 4° with primary antibodies (SCP1 (Novus), SCP3 (Novus and gift of S. West), MLH1 (Calbiochem), RAD51 (Lab Vision), DMC1 (Santa Cruz), and γ-H2AX (Upstate)) diluted in 10x block. Slides were rinsed and incubated 2 hours at room temperature with secondary antibodies (AlexaFluor-488 and -594 goat anti-mouse and goat anti-rabbit IgG (Molecular Probes)) diluted in 10x block. Slides were rinsed, soaked briefly in 100 ng/ml DAPI (Sigma), rinsed, and allowed to dry in the dark. Coverslips were mounted with antifade media (1 mg/ml p-phenylenediamine, 1x PBS, 80% glycerol) and sealed with nail polish.

For diakinesis spread preparations, testes were harvested and cell suspensions were prepared as described above. Cells were pelleted at low speed and resuspended dropwise in 1 ml hypotonic solution (0.075 M KCl), pre-warmed to 37 degrees. Hypotonic solution was brought up to 5 ml and cells incubated at 37 degrees for 15 minutes. 2.5 ml ice-cold fixative was added (75% methanol, 25% acetic acid), and cells pelleted by centrifugation at low speed. Supernatant was aspirated, cells resuspended in residual fluid, and rinsed in 5 ml cold fixative. Pelleted cells were resuspended in ∼500 ul fixative and dropped onto humid slides, rinsed with 1 ml fix, and dried over humid paper towels. Slides were stained with Giemsa (Sigma) and cover slips were mounted with Permount (Fisher).

### Spermatocyte Analyses and Measurements

For analysis of immunologically stained spermatocyte spreads, morphology of the SC and configuration of the sex bivalent was used for prophase staging. Cells with obvious abnormalities, like severe fragmentation of SCs, were excluded from analyses since the frequency of these cells did not significantly differ between mutants and controls. At least three mutant and control pairs were employed for each of these studies.

To assay crossovers, the mismatch repair protein MLH1 was utilized. The number of MLH1 foci was scored for each pachytene spermatocyte containing 19 fully synapsed autosomal SCs, one partially synapsed XY bivalent and a minimum of 18 foci (excluding the sex bivalent foci). This threshold was chosen based on the observation that cells containing ∼18 or more foci consistently exhibited MLH1 foci signals that significantly exceeded background noise levels. Although cells with less than 18 foci were frequently observed, non-linear manipulation of signal was often required to produce sufficient contrast for analysis. Double exchange bivalents from these analyses were used to examine interference patterns. Measurements to determine total SC lengths and inter-MLH1 distances were made in Volocity (Improvision). Measurements were taken from 3 different mice per genotype, with 137 bivalents analyzed from *Zip4h^−/Y^* mice and 190 bivalents from *Zip4h^+/Y^*in total.

To assess resolution of DSB repair intermediates, the numbers of DMC1 foci in pachytene spermatocytes were quantified. Only cells containing 19 fully formed autosomal bivalents were scored, and only foci co-localized with the SC were counted.

To examine chiasmata in diakinesis phase spermatocytes, chromosome spreads were prepared from 2 mutant and control pairs. Only spreads where a total of 20 bivalents could be identified, or where bivalents plus univalents were equal to 20 homolog pairs, were analyzed. Spreads with missing chromosomes, or cells with overlapping bivalents were excluded from analysis.

### Histological Sample Preparation and Analysis

Testes were harvested from adult males between the ages of 8 and 16 weeks. For immunohistochemical and histological staining, testes were fixed overnight at 4° in Bouin's solution (Sigma) and washed 3 days in 3 changes of 70% ethanol. Tissues were processed for paraffin embedding, 8 um sections were prepared, and samples were auto-stained at the SKI Molecular Cytology Facility.

To determine which cell populations expressed ZIP4H in testes, serial sections were stained with anti-ZIP4H antibody (goat anti-mouse-ZIP4H (R & D Systems)) and periodic acid Schiff's (PAS) histology stain. Staging of tubules and identification of sub-populations were carried out using the criteria described by Russell et al. [39].

For laggard chromosome analyses, stage XII tubules from PAS stained testis sections were identified and MI cells with metaphase chromosomes and spindles were assessed for the presence of laggard chromosomes. 3 mice were analyzed per genotype with a minimum of 140 total metaphase spindles assessed per genotype.

For TUNEL staining, testes from 2 mutant and control pairs were fixed overnight in 4% PFA, transferred to 70% ethanol, processed as above for preparation of sections, and stained with the in-situ cell death kit (Roche) following the manufacturers instructions with the exception of BrdU labeling was carried out for 15 minutes. Stage XII tubules were identified in the DAPI channel followed by tabulation of TUNEL positive cells in the FITC channel.

### Microscopy and Imaging

Fluorescently labeled samples were viewed on a Zeiss Axiovert epifluorescence microscope and imaged with a Hammamatsu CCD camera. Histology samples were viewed on an Olympus IX60 microscope and imaged with a Q-color 5 CCD camera. Volocity (Improvision) was used for image acquisition and analysis. Slides and images were scored blind as to the status of the mice (control or mutant).

### Statistical Analyses

In order to avoid making assumptions regarding whether data were normally distributed, non-parametric statistical methods were employed. The Mstat software version 4.01 (available at [87]) was utilized for Wilcoxan rank-sum (Mann-Whitney) analysis of DMC1, MLH1 and TUNEL data, and Mantel-Haenszel analysis of laggard and achiasmate chromosome data. With respect to the interference data, a table was constructed containing the proportion of SCs with inter-crossover distances falling below each 10^th^ percentile of the total SC length for the purpose of comparison between mutant and control proportions by category. Visual inspection of the data indicated that the proportion of mutant SCs containing short inter-crossover distances was consistently higher in the mutant. Chi-squared analysis was carried out to assess differences between the proportions.

For maximum likelihood fitting of interference data to the Gamma Distribution the Wessa Gamma Distribution calculator was utilized [57]. PRISM was used to produce overlay graphs of interference distribution and best fit gamma-distribution curves.

## Supporting Information

Text S1 Supporting Text.
(0.06 MB DOC)

Table S1 Zip4h expression in spermatogonia and spermatocytes.
(0.03 MB DOC)

Table S2 Synaptonemal complex aberrations.
(0.03 MB DOC)

Figure S1 Northern blot, generation of targeting construct, targeting, deletion transcript, genotype PCRs and NBS1 interaction. Full length *Zip4h* cDNA was labeled and used for Northern blot analysis of transcripts from multiple human tissues (A). Exon 3 of the wild type *Zip4h* locus (B) was targeted for conditional deletion. The targeting construct was electroporated into ES cells to generate the *Zip4h^ind^* locus (C). The *Zip4h^−^* locus (D) is generated through mating of the inducible strain to *Cre*-expressing transgenic mice. Orange triangles, LoxP sites; purple triangles, FRT sites. Southern blot analysis of BamHI (E) or StuI (F) digested genomic DNA from ES cell clones generated from the *Zip4h^ind^* targeting construct (see B, C for locus probes), indicated configuration of the *Zip4h^ind^* allele on the 5′ and 3′ ends of the locus, respectively (5′ LoxP- indicates integrant lacking the 5′ LoxP site). Targeted ES cells (G) were transfected with a *Cre* expression construct. Exon 3 deletion is expected to produce a variant transcript in which exon 2 is spliced to exon 4, resulting in a frameshift and termination codon (I). A PCR strategy was devised to assess exon 3 deletion (H) and was used for RT-PCR analysis of the transcripts (J). Sequencing of the PCR product from J indicated that the predicted exon 2 to 4 spliced transcript was present (I). Genotype PCR (K) of *Zip4h^+/Y^, Zip4h^ind^* and *Zip4h^−/Y^* mice (see Figure B–D for locus maps and primers). IP-Western blots performed on 293T cells transiently expressing MYC-tagged human ZIP4H indicate a small quantity of endogenous NBS1 interacts with the recombinant ZIP4H (L).
(1.86 MB TIF)

Figure S2 *Zip4h* expression in spermatogonia and spermatocytes. Wildtype testis sections were immunohistochemically labeled with ZIP4H antibody. Representative images from all 12 stages of the sermatogenic cycle are shown (I-XII). Abbreviations are used to label various cell populations: Sert, sertoli cell; SG, sermatogonia; PL, pre-leptotene; L, leptotene; Z, zygotene; P, pachytene; SB, sex body; D, diplotene; Ana, anaphase; RS, round spermatid; ES, elongating spermatid.
(4.95 MB TIF)

Figure S3 Spermatocyte spreads from *Zip4h^−/Y^* mice appear normal. Spermatocytes spreads from *Zip4h^−/Y^* mice were immunofluorescently labeled with SCP3 (red) and SCP1 (green) to assess axial element formation and synapsis. Representative cells are shown arrayed from left to right in order of advancing meiotic stage: leptotene (A), zygotene (B–D), pachytene (E–G) and diplotene (H, I). Wherever applicable, the XY bivalent is indicated by *. In G, a dotted line demarks the boundary of a late pachytene cell from a neighboring cell.
(8.16 MB TIF)

Figure S4 γ-H2AX foci on *Zip4h^−/Y^* bivalents. Representative γ-H2AX stained pachytene stage spermatocyte spread from a *Zip4h^−/Y^* mouse (A). Co-labeling with γ-H2AX, SCP3, MLH1 and RAD51 indicates that MLH1 and RAD51 foci on Z*ip4h^−/Y^* bivalents (B and D, merged images) also contained the DSB marker γ-H2AX (C and E, same images as B and D showing SCP3 and γ-H2AX alone). Some bivalents undergoing crossover formation also contained γ-H2AX foci that did not stain positive for RAD51 (yellow arrows, D and E).
(4.00 MB TIF)
