# Supplementary material for: ZIP4H (TEX11) Deficiency in the Mouse Impairs Meiotic Double Strand Break Repair and the Regulation of Crossing Over
Source: PLoS Genet. 2008 Mar 28;4(3):e1000042. doi: 10.1371/journal.pgen.1000042 (PMC2267488; doi:10.1371/journal.pgen.1000042)
Supplement: Text S1 — Supporting Text. (0.06 MB DOC) [file pgen.1000042.s001.doc]

**Meiotic Prophase stages (in chronological order)**

*Leptotene*- Stage in which DSBs are formed and processed, and strand exchange intermediates begin to appear. Homolog pairing and axial element assembly is initiated.

*Zygotene*- Repair of DSBs is ongoing. The number of strand exchange intermediates declines as cells reach late zygotene. Elaboration of axial elements continues and synapsis between homologous chromosomes is initiated in early zygotene. Synapsis nears completion at late zygotene stages.

*Pachytene-* Strand exchange intermediates are resolved and crossovers are formed. Homologs are completely synapsed during pachytene with the exception of the heterologous axes of the sex chromosomes in males. These chromosomes become sequestered in a proteinacious chromatin domain, termed the sex-body, during pachytene in male meiocytes.

*Diplotene*- Homologs desynapse everywhere except at centromeres. Homologs remain associated via crossovers and sister chromatid cohesion.

*Diakinesis/Metaphase I*- Chromosomes condense and attachment of homolog centromeres to opposite spindle poles allow for bivalent alignment on the metaphase plate.

# Supplemental Methods

## Northern blot

Full length human *Zip4h* cDNA was used for probe generation and was blotted against a human Multiple Tissue Northern Blot purchased from Clontech. Blot was pre-hybridized for 30 minutes with blotting buffer supplied by the manufacturer at 68°. The probe was boiled, added to the hybridization buffer, and allowed to hybridize for 1 hour at 68°. The blot was washed according to manufacturers instructions and exposed to a phosphorimager screen.

## Gene targeting

The *Zip4h^ind^* targeting vector was constructed using the recombineer method of Liu et al. [1]. Targeting was carried out via electroporation into AC1 ES cells (gift of G. Oltz and E. Rhuley). Selection, clonal expansion and analysis were performed using previously published procedures [2]. Chimeras were generated at the SKI Transgenic Facility using standard methods, and female offspring were mated with *CAG-Cre* transgenic mice [3] to generate the *Zip4h^-^* allele. Details of the targeting vector construction and genotyping will be provided upon request.

**References**

1. Liu P, Jenkins NA, Copeland NG (2003) A highly efficient recombineering-based method for generating conditional knockout mutations. Genome Res 13: 476-484.

2. Luo G, Yao MS, Bender CF, Mills M, Bladl AR, et al. (1999) Disruption of mRad50 causes embryonic stem cell lethality, abnormal embryonic development, and sensitivity to ionizing radiation. Proc Natl Acad Sci U S A 96: 7376-7381.

3. Sakai K, Miyazaki J (1997) A transgenic mouse line that retains Cre recombinase activity in mature oocytes irrespective of the cre transgene transmission. Biochem Biophys Res Commun 237: 318-324.
