# Supplementary material for: ZIP4H (TEX11) Deficiency in the Mouse Impairs Meiotic Double Strand Break Repair and the Regulation of Crossing Over
Source: PLoS Genet. 2008 Mar 28;4(3):e1000042. doi: 10.1371/journal.pgen.1000042 (PMC2267488; doi:10.1371/journal.pgen.1000042)
Supplement: Table S1 — Zip4h expression in spermatogonia and spermatocytes. (0.03 MB DOC) [file pgen.1000042.s002.doc]

| **Cell Type** | **Expression** |
| --- | --- |
| A-Intermediate Spermatogonia | - |
| B Spermatogonia | +/- |
| Pre-Leptotene | + |
| Leptotene | + |
| Zygotene | ++ |
| Pachytene | +/- |
| Diplotene | - |
