# Supplementary material for: ZIP4H (TEX11) Deficiency in the Mouse Impairs Meiotic Double Strand Break Repair and the Regulation of Crossing Over
Source: PLoS Genet. 2008 Mar 28;4(3):e1000042. doi: 10.1371/journal.pgen.1000042 (PMC2267488; doi:10.1371/journal.pgen.1000042)
Supplement: Table S2 — Synaptonemal complex aberrations. (0.03 MB DOC) [file pgen.1000042.s003.doc]

| **Genotype** | **% Cells with SC**  **Aberrations** | | | **% Autosomes with SC Aberrations** | | | **N** |
| --- | --- | --- | --- | --- | --- | --- | --- |
|  | **Gap or Fragment** | **Fusion** | **Other** | **Gap or Fragment** | **Fusion** | **Other** | **Cells / Autosomes** |
| *Zip4h^+/Y^* | 26.9 | 3.85 | 3.85 | 1.41 | 0.20 | 0.20 | 52/990 |
| *Zip4h^-/Y^* | 26 | 2 | 2 | 1.37 | 0.11 | 0.11 | 50/951 |
