# Supplementary figures and images for: ZIP4H (TEX11) Deficiency in the Mouse Impairs Meiotic Double Strand Break Repair and the Regulation of Crossing Over
Source: PLoS Genet. 2008 Mar 28;4(3):e1000042. doi: 10.1371/journal.pgen.1000042 (PMC2267488; doi:10.1371/journal.pgen.1000042)

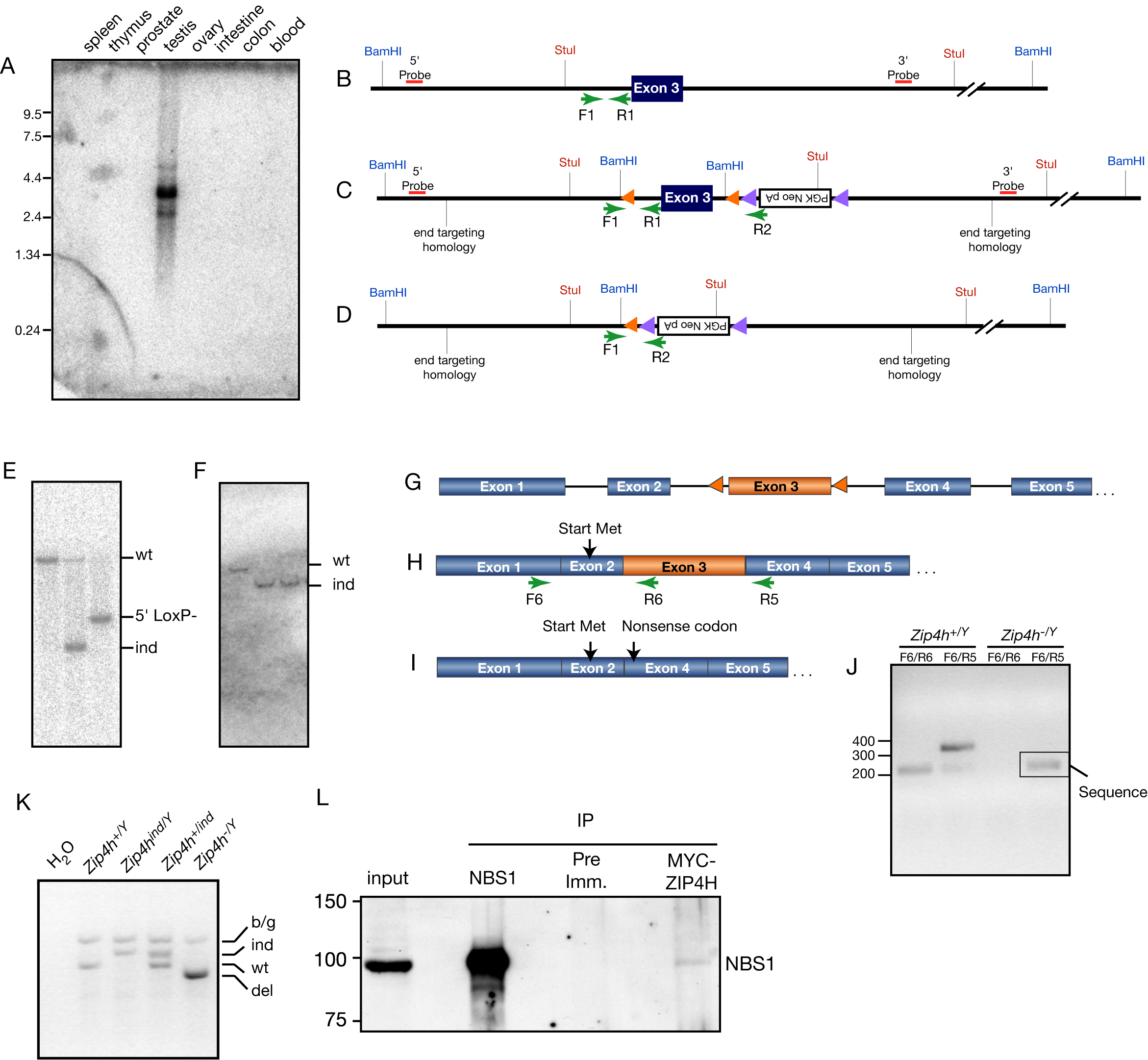

Supplement: Figure S1 — Northern blot, generation of targeting construct, targeting, deletion transcript, genotype PCRs and NBS1 interaction. Full length Zip4h cDNA was labeled and used for Northern blot analysis of transcripts from multiple human tissues (A). Exon 3 of the wild type Zip4h locus (B) was targeted for conditional deletion. The targeting construct was electroporated into ES cells to generate the Zip4hind locus (C). The Zip4h− locus (D) is generated through mating of the inducible strain to Cre-expressing transgenic mice. Orange triangles, LoxP sites; purple triangles, FRT sites. Southern blot analysis of BamHI (E) or StuI (F) digested genomic DNA from ES cell clones generated from the Zip4hind targeting construct (see B, C for locus probes), indicated configuration of the Zip4hind allele on the 5′ and 3′ ends of the locus, respectively (5′ LoxP- indicates integrant lacking the 5′ LoxP site). Targeted ES cells (G) were transfected with a Cre expression construct. Exon 3 deletion is expected to produce a variant transcript in which exon 2 is spliced to exon 4, resulting in a frameshift and termination codon (I). A PCR strategy was devised to assess exon 3 deletion (H) and was used for RT-PCR analysis of the transcripts (J). Sequencing of the PCR product from J indicated that the predicted exon 2 to 4 spliced transcript was present (I). Genotype PCR (K) of Zip4h+/Y, Zip4hind and Zip4h−/Y mice (see Figure B–D for locus maps and primers). IP-Western blots performed on 293T cells transiently expressing MYC-tagged human ZIP4H indicate a small quantity of endogenous NBS1 interacts with the recombinant ZIP4H (L). (1.86 MB TIF) [file pgen.1000042.s004.tif]

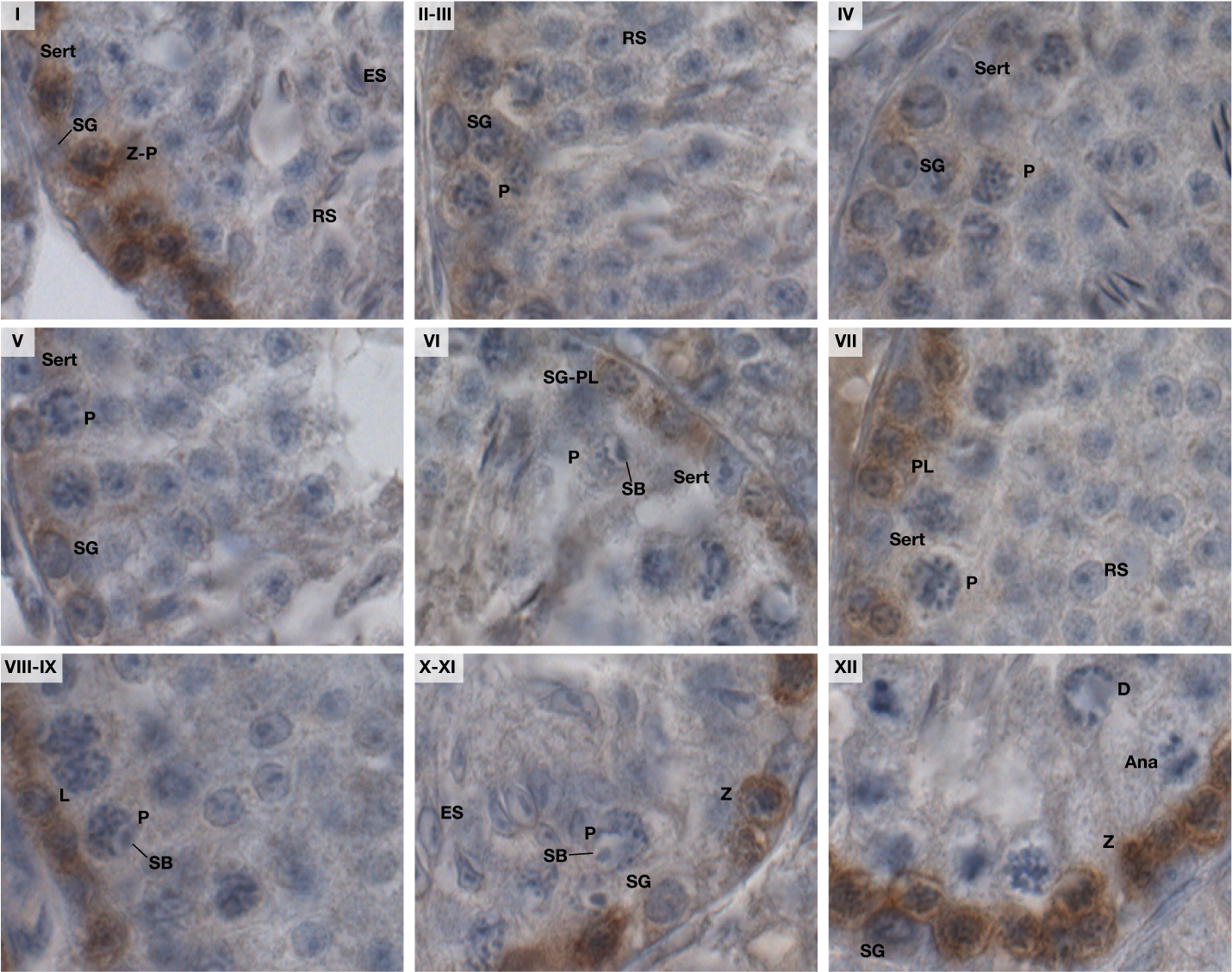

Supplement: Figure S2 — Zip4h expression in spermatogonia and spermatocytes. Wildtype testis sections were immunohistochemically labeled with ZIP4H antibody. Representative images from all 12 stages of the sermatogenic cycle are shown (I-XII). Abbreviations are used to label various cell populations: Sert, sertoli cell; SG, sermatogonia; PL, pre-leptotene; L, leptotene; Z, zygotene; P, pachytene; SB, sex body; D, diplotene; Ana, anaphase; RS, round spermatid; ES, elongating spermatid. (4.95 MB TIF) [file pgen.1000042.s005.tif]

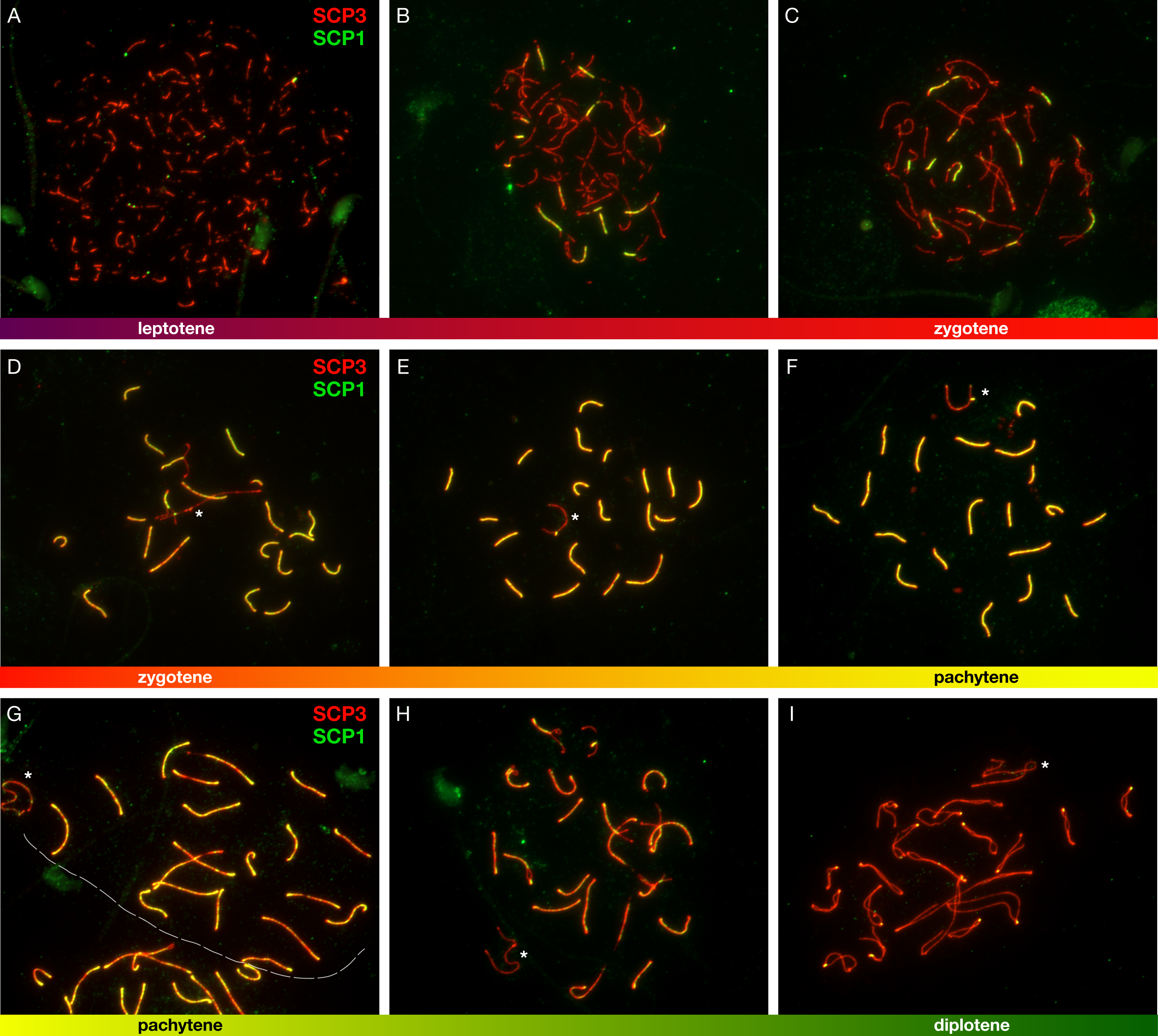

Supplement: Figure S3 — Spermatocyte spreads from Zip4h−/Y mice appear normal. Spermatocytes spreads from Zip4h−/Y mice were immunofluorescently labeled with SCP3 (red) and SCP1 (green) to assess axial element formation and synapsis. Representative cells are shown arrayed from left to right in order of advancing meiotic stage: leptotene (A), zygotene (B–D), pachytene (E–G) and diplotene (H, I). Wherever applicable, the XY bivalent is indicated by *. In G, a dotted line demarks the boundary of a late pachytene cell from a neighboring cell. (8.16 MB TIF) [file pgen.1000042.s006.tif]

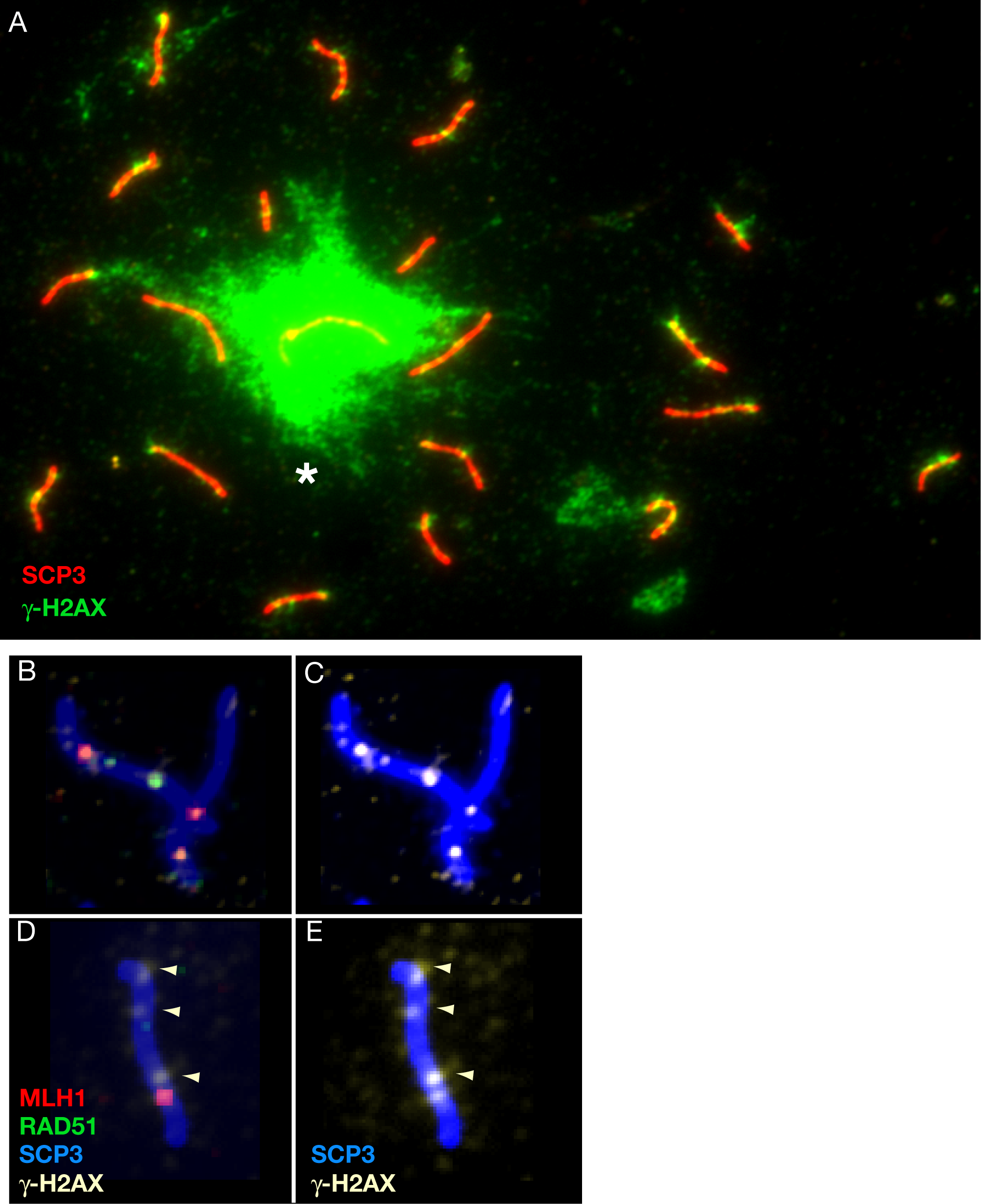

Supplement: Figure S4 — γ-H2AX foci on Zip4h−/Y bivalents. Representative γ-H2AX stained pachytene stage spermatocyte spread from a Zip4h−/Y mouse (A). Co-labeling with γ-H2AX, SCP3, MLH1 and RAD51 indicates that MLH1 and RAD51 foci on Zip4h−/Y bivalents (B and D, merged images) also contained the DSB marker γ-H2AX (C and E, same images as B and D showing SCP3 and γ-H2AX alone). Some bivalents undergoing crossover formation also contained γ-H2AX foci that did not stain positive for RAD51 (yellow arrows, D and E). (4.00 MB TIF) [file pgen.1000042.s007.tif]
